# RNAi-mediated gene knockdown by microinjection in the model entomopathogenic nematode *Heterorhabditis bacteriophora*

**DOI:** 10.1186/s13071-016-1442-4

**Published:** 2016-03-18

**Authors:** Ramesh Ratnappan, Jonathan Vadnal, Melissa Keaney, Ioannis Eleftherianos, Damien O’Halloran, John M. Hawdon

**Affiliations:** Department of Microbiology Immunology and Tropical Medicine, George Washington University Medical Center, Washington, DC 20037 USA; Department of Biological Sciences, George Washington University, Science and Engineering Hall, suite 6000, 800 22nd Street NW, Washington, DC 20052 USA; Institute for Neuroscience, George Washington University, 636 Ross Hall, 2300 I Street NW, Washington, DC 20052 USA

**Keywords:** *Heterorhabditis bacteriophora*, Microinjection, RNAi, Gene knockdown, qRT-PCR

## Abstract

**Background:**

Parasitic nematodes threaten the health of humans and livestock and cause a major financial and socioeconomic burden to modern society. Given the widespread distribution of diseases caused by parasitic nematodes there is an urgent need to develop tools that will elucidate the genetic complexity of host-parasite interactions. *Heterorhabditis bacteriophora* is a parasitic nematode that allows simultaneous monitoring of nematode infection processes and host immune function, and offers potential as a tractable model for parasitic nematode infections. However, molecular tools to investigate these processes are required prior to its widespread acceptance as a robust model organism. In this paper we describe microinjection in adult *H. bacteriophora* as a suitable means of dsRNA delivery to knockdown gene transcripts.

**Methods:**

RNA interference was used to knockdown four genes by injecting dsRNA directly into the gonad of adult hermaphrodite nematodes. RNAi phenotypes were scored in the F1 progeny on the fifth day post-injection, and knockdown of gene-specific transcripts was quantified with real-time quantitative RT-PCR (qRT-PCR).

**Results:**

RNAi injection in adult hermaphrodites significantly decreased the level of target transcripts to varying degrees when compared with controls. The genes targeted by RNAi via injection included *cct-2*, *nol-5*, *dpy-7*, and *dpy-13*. In each case, RNAi knockdown was confirmed phenotypically by examining the progeny of injected animals, and also confirmed at the transcriptional level by real-time qRT-PCR.

**Conclusions:**

Here we describe for the first time the successful use of microinjection to knockdown gene transcripts in *H. bacteriophora.* This technique can be used widely to study the molecular basis of parasitism.

## Background

Diseases caused by parasitic nematodes are a major concern, resulting in human health and socioeconomic consequences [1, 2]. It is estimated that more than half of the human population is infected with gastrointestinal nematodes alone [3, 4], and around 20 species routinely cause disease [5]. Parasitic nematodes are also a concern to the livestock industry, as they cause diseases and financial loss estimated in the tens of millions of dollars per year [6–8]. Parasitic nematode control in humans and livestock is limited to periodic treatment with anthelmintics, but development of resistance to the commonly used drugs is an ongoing problem, limiting efficacy and requiring continual development of new anthelmintics [9, 10]. The search for new drugs, as well as alternate novel control methods such as vaccines, requires comprehensive knowledge of the host-parasite interaction.

An important interaction between the parasite and its potential host is the active avoidance of the host immune system by the parasite [11]. Understanding this interaction at the molecular level provides useful insight into pathogen virulence and host immunity [12, 13]. Advances in understanding these mechanisms require powerful molecular tools to investigate gene function, which are unavailable for most parasitic nematodes. Discovery of RNA interference (RNAi) in *Caenorhabditis elegans* was a major step forward in the analysis of gene function [14]. RNAi is the RNA-induced silencing of gene-specific mRNA targets [15]. In *C. elegans* RNAi is performed either by soaking in double-stranded RNA (dsRNA), feeding the nematodes with bacteria expressing dsRNA or by injecting the dsRNA into the gonad of mature adult hermaphrodite nematodes [16]. In *C. elegans*, RNAi has been successfully used to knockdown almost all the genes in the genome [17]. Several studies have adapted this technique to parasitic nematodes in attempts to knockdown genes in order to understand host-pathogen interactions [18, 19]. RNAi based gene knockdown has been tested, with mixed success, in both plant and animal parasitic nematodes. Animal parasitic nematodes in which RNAi has been tested include *Nippostrongylus brasiliensis* [20, 21], *Brugia malayi* [22, 23], *Onchocerca volvulus* [24], *Litomosoides sigmodontis* [25], *Ascaris suum* [26, 27], *Trichostrongylus colubriformis* [28], *Haemonchus contortus* [29–31], *Ostertagia ostertagi* [32], *Teladorsagia circumcincta* [33], *Trichinella spiralis* [34], *Heligmosomoides polygyrus* [35] and *Heterorhabditis bacteriophora* [36, 37].

RNAi experiments conducted in parasitic nematodes have been performed by soaking or feeding the dsRNA. However, significant variability in both detecting the desired phenotype and reduction in target gene transcripts is common [38, 39]. RNAi in *Heligmosomoides polygyrus* has failed to produce any observable knockdown [35]. Considerable variation was observed between genes knocked down in *Haemonchus contortus;* in one study, gene knockdown occurred only in genes that are expressed in tissues that come in direct contact with dsRNA, such as the intestine [40]. Another study found that only administration of dsRNA by feeding resulted in expected phenotypes [31]. Nearly complete knockdown of several target genes has also been reported in *H. bacteriophora* when dsRNA is administered by soaking [36]. This disparity in observed phenotypes cannot be explained solely by differences in the RNAi machinery existing in parasitic nematode species. Analysis of the RNAi machinery has revealed that most parasites contain at least the minimal requirements for a functional RNAi pathway. Noticeably absent are the genes involved in amplification, uptake and spread of dsRNA, including SID-2, RSD-2 and RSD-6 [41]. This variation in RNAi efficacy has hampered investigations of parasitic gene function. Hence, there is a need to increase the efficacy and reliability of gene knockdown by RNAi.

*Heterorhabditis bacteriophora* is an entomopathogenic nematode, a member of the family Heterorhabditidae in which all nematodes are obligate parasites [42]. It is grouped in the Eurhabditis clade that includes the hookworms *Ancylostoma ceylanicum, A. duodenale* and *Necator americanus* that infect humans and other vertebrates. The Eurhabditis clade also includes the well-studied model nematode *C. elegans* [43]. The divergence time between *H. bacteriophora* and *C. elegans* is estimated to be between 86 and 331 MYA [44]. *H. bacteriophora* has several important similarities with *C. elegans*. These include small size, transparency, ease of in vitro culture, short generation time, both hermaphroditic and gonochoristic reproduction, and an annotated genome with about the same number of genes [44]. Moreover, the infective stage of *H. bacteriophora* and strongylid nematodes is an obligate arrested third larval stage (L3) that is similar to the facultative arrested dauer stage of *C. elegans*. These similarities, as well as its close phylogenetic relationship with *C. elegans* and other important parasites of humans and animals, make *H. bacteriophora* a potentially excellent model for molecular studies of nematode infection mechanisms. The only free living stage of *H. bacteriophora* is a non-feeding, developmentally arrested infective juvenile (IJ) stage. The other stages (i.e. L1, L2, L3, L4 and adult) develop inside the host. The developmentally arrested IJs can survive for months in the soil while seeking a host. After entry into the insect host, IJs regurgitate their endosymbiotic bacterium *Photorhabdus luminescens* into the insect haemocoel, where it replicates and contributes to the inevitable death of the host within 24–72 h [42, 45]. IJs resume their development in the host, developing only into phenotypically female hermaphrodites. Males, females and hermaphrodites are produced in approximately equal numbers in subsequent generations in the presence of abundant food [46]. The nematodes reproduce for another 2–3 generations feeding on the insect cadaver. However, nutrient limitation and accumulation of density limiting pheromones [47] impedes further progression of the life-cycle and causes mass production of IJs. IJs colonized with *P. luminescens* then exit the cadaver in large numbers into the external environment. These IJs search for and infect other insect hosts to continue the cycle [48].

Two previous studies in *H. bacteriophora* have demonstrated that delivery of dsRNA by soaking can successfully knockdown gene transcripts [36, 37]*.* However, dsRNA delivery either by feeding or by microinjection in *H. bacteriophora* has not been reported. Very few studies have successfully demonstrated the use of microinjection as a method of introducing dsRNA in parasitic nematodes [26, 49], yet RNAi via microinjection represents the most reliable and least variable method of gene silencing in *C. elegans* for most targets [16]. In the present study we describe RNAi in *H. bacteriophora* by injecting dsRNA into the gonads of the adult hermaphrodite. We show that gene function can be successfully knocked down in the progeny of injected nematodes by this technique. RNAi by microinjection provides an alternative approach that can be widely used to study the function of genes involved in parasitism of *H. bacteriophora*.

## Methods

### Nematodes

*H. bacteriophora* strain TT01 was kindly provided by Dr. David Clarke (University College Cork, Ireland). Nematode stocks were maintained in the lab by infecting the Greater Wax Moth larvae (*Galleria mellonella*) with IJs [50]. IJs emerging from white traps [51] were propagated on lawns of *P. luminescens* [36] to raise young hermaphrodites for injection.

### Primer design

Primers for dsRNA were designed to target ~500 base pair exonic regions of *H. bacteriophora* DNA. In order to identify possible exons, protein BLASTs were performed to identify regions of similarity between *H. bacteriophora* and *C. elegans* for *dpy-7* and *dpy-13* genes. Primers for regions of interest were determined using Primer3 [52], selecting for an optimum product length of 500 base pairs, *T*_m_ of 60 °C, and primer length of 22 nucleotides. The primer pair for Green Fluorescent Protein (GFP) was designed using the same settings in Primer3. T7 sites (TAATACGACTCACTATAGGG) were added to the 5′ ends of each primer to allow for in vitro transcription.

### dsRNA synthesis

Genomic DNA was isolated from frozen pellets of ~50,000 *H. bacteriophora* infective juveniles. An IJ pellet was resuspended in 50 μl of lysis buffer (50 mMKCl, 0.05 % (w/v) gelatin, 10mMTris-HCl pH 8.2, 0.45 % Tween 20, 60 μg/ml Proteinase K, 2.5 mM MgCl_2_) and placed at −80 °C for 30 min. The solution was then warmed to room temperature and incubated at 60 °C for 2 h, with vortexing every 15 min. Proteinase K was denatured by incubating the homogenized tissue for 15 min at 95 °C. The sample was then cooled to 4 °C and centrifuged at 3,400 × g for 1 min. The resulting supernatant was used as template for subsequent PCR. A 50 μl PCR reaction was carried out using ChoiceTaq Mastermix (Denville Scientific, South Plainfield, NJ, USA) with 200 ng of template DNA, 0.2 μM of each primer, and the manufacturer’s suggested cycling conditions. PCR reactions were analyzed on a 1.2 % agarose gel to verify that the reactions produced single bands of the predicted size.

Five μl of the PCR reaction was used for in vitro transcription using the Ambion Megascript T7 Kit (Thermo Fisher Scientific, Waltham, MA, USA). Reactions were carried out following the manufacturer’s instructions and incubated for 16 h at 37 °C. In vitro transcription reactions were cleaned up using the AmbionMegaclear Kit (Thermo Fisher, Waltham, MA, USA) followed by ammonium acetate/ethanol precipitation to concentrate the dsRNA. Pelleted dsRNA was suspended in 10 μl RNase-free water, quantified using a NanoDrop spectrophotometer, and the quality assessed by separating the dsRNA on a 1.2 % agarose gel.

### RNAi by injection

dsRNA was injected into the gonad of adult hermaphrodite nematodes as described for *C. elegans* [53]. Young adults were obtained by placing IJs collected from white traps on aNA + chol agar plate (3 g yeast extract, 5 g peptone, 12 g agarose per liter with 2 ml of 5 mg/ml cholesterol added after autoclaving) growing *P. luminescens* bacteria for 68 h at 27 °C. dsRNA was injected at a concentration of 6 μg/μl for *cct-2* and 4 μg/μl for*nol-5, dpy-13* and *dpy-7*. The injected nematodes were removed from the injection pad with a pipette using 1xPBS and placed on a rescue plate. After 1 h the nematodes were picked to a new plate seeded with fresh *P. luminescens* and maintained at 27 °C. Three batches of nematodes were used, representing three different biological replicates each with 15 nematodes. The progeny of the injected hermaphrodite were screened on the fifth day after injection. Images were taken on a stereomicroscope (LeicaS6 D, Leica, Germany).

### RNA extraction and qRT-PCR

Total RNA was extracted in TRIzol (Thermo Fisher). Briefly, 20 worms for *Hb-cct-2* and *Hb-nol-5* and 2 worms for *Hb-dpy-7* and *Hb-dpy-13* were picked from the *P. luminescens* plate into a 1.5 ml tube with M9 buffer (3 g KH2PO4, 6 g Na2HPO4, 5 g NaCl, 1 ml 1 M MgSO4, H2O to 1 l). Nematodes were washed with M9 two more times after which 250 μl of Trizol was added. The tubes were stored at −80 °C. On the day of RNA extraction, the tubes were thawed and vortexed at maximum speed for 30 min at 4 °C. RNA was extracted according to the manufacturer’s instructions. Total RNA was treated with DNase I, Amplification Grade (Thermo Fisher). RNA was then converted to cDNA with Verso™ cDNA Kit (Thermo Fisher). Real-time quantitative reverse transcription PCR (qRT-PCR) was performed in a 96-well Bio-Rad CFX96 RealTime PCR System (Bio-Rad, Inc., Hercules, CA, USA). PCR reactions were done in 96-well optical reaction plates (Bio-Rad, Hercules, CA, USA) using Agilent Brilliant II SYBR® Green QPCR Master Mix. A 20 μL PCR reaction was set up in each well with 10 μL Brilliant II SYBR Green QRT-PCR master mix, 1/20th of the converted cDNA and 25 μM primers. To quantify the efficiency of the primers, a standard curve was constructed with serial dilutions of gene-specific PCR products that were obtained by amplifying cDNA from adults collected 72 h after plating as IJs. The amplification efficacy (E = 10^1/-slope^-1) for each primer was calculated from the slope generated by the standard curve. The primer sets used had efficiency between 90–100 %. Relative quantification of the amplified gene was done by delta-delta Ct method [54]. Large ribosomal subunit L32 protein (*rpl-32*) was used as an internal control. Expression of *Hba-rpl-32* was shown to be stable in three different life stages of *H. bacteriophora* (IJs and 48 h and 72 h developing nematodes derived from IJs) before it was used as an internal reference gene. For every gene at least three independent biological samples were tested, each with three technical replicates. Primers used in this study are listed in Table 1.

**Table 1:** Primer sequences used for qRT-PCR

Primer name	Sequence
*dpy-13Fwd*	AGCCCGGAGCTAAAGGTAAC
*dpy-13Rev*	TACGAGTCATCAATGGCACA
*dpy-7-Fwd*	GGTAGACCAGGTCGTCCAGT
*dpy-7-Rev*	ACCAGGCAAACCAGGACTT
*rpl-32-Fwd*	ATCGGATAGATACCACCGCC
*rpl-32-Rev*	TTGTGGGCATAGCACGC

## Results and discussion

We tested the efficacy of dsRNA delivery by microinjection on four *H. bacteriophora* genes with known RNAi phenotypes. The *C. elegans cct-2* (T21B10.1) gene encodes a component of eukaryotic T-complex chaperonin and is expressed in most tissues. It is required for proper folding of proteins including actin, tubulin and cyclin [55–57]. In *C. elegans*, RNAi of *cct-2* causes sterility, embryonic lethality and protruding vulva [17, 58]. The *C. elegans nol-5* is an ortholog of human NOP58 (NOP58 ribonucleoprotein) and is involved in reproduction, embryo development, and larval development. Knockdown of *nol-5* in *C. elegans* causes larval arrest, multivulva phenotype, sterility and slow growth [58, 59]. Ciche et al. [36] showed previously that knockdown of *Hba-cct-2* and *Hba-nol-5*by soaking eggs in dsRNA also causes sterility in *H. bacteriophora*, as evidenced by the absence of visible germline and defective gonad.

**Fig. 1:**
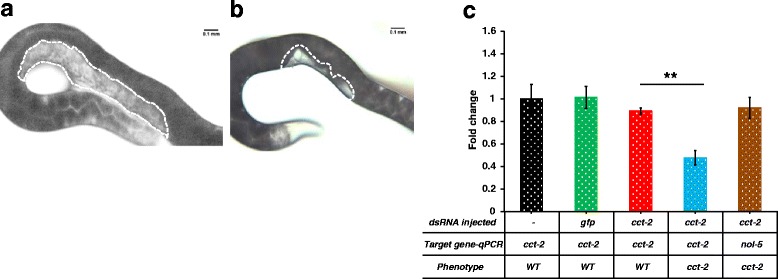
RNAi mediated phenotype and transcript changes in *H. bacteriophora* injected with *cct-2* dsRNA. Adult *H. bacteriophora* hermaphrodites that were injected with *cct-2* dsRNA produced progeny with no germline and empty gonad. **a**. Progeny of non-injected *H. bacteriophora*. The area with white dotted lines indicates the position of the gonads. **b**. Progeny of *H. bacteriophora* injected with *cct-2* dsRNA. **c**. Expression of *cct-2* gene in the progeny of injected worms. The y-axis represents the fold change in mRNA expression in the progeny of *H. bacteriophora* hermaphrodites. The mRNA levels are normalized to *cct-2* expression in the progeny of non-injected hermaphrodites (*black bar*). The green bar represents *cct-2* levels in progeny of *gfp* injected worms. The red bar represents *cct-2* levels in a phenotypically wild type sibling, and the blue bar represents the *cct-2* levels in worms with empty gonads. To control for off-target effects, expression of an unrelated gene (*nol-5*) in worms with empty gonads was determined (*brown bar*). The graph was obtained by combining data from at least three independent biological replicates. Error bars indicate the standard error of the mean. Asterisks depict the statistical significance of the observed differences in unpaired, two-tailed t-tests with *P*-values < 0.001 (**)

**Fig. 2:**
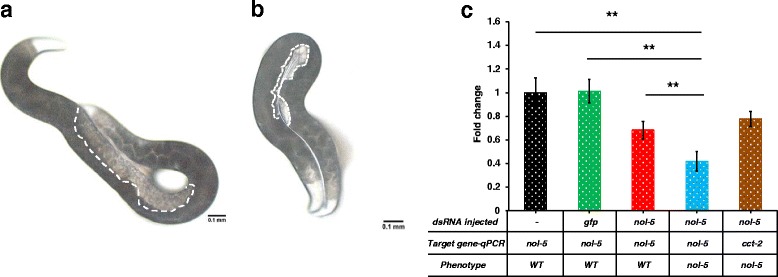
RNAi mediated phenotype and transcript changes in *H. bacteriophora* injected with *nol-5* dsRNA. Adult *H. bacteriophora* hermaphrodites that were injected with *nol-5* dsRNA produced progeny with no germline and empty gonad. **a**. Progeny of non-injected *H. bacteriophora*. The area with white dotted lines indicates the position of the gonads. **b**. Progeny of *H. bacteriophora* injected with *nol-5*dsRNA. **c**. Expression of *nol-5* gene in the progeny of *nol-5* dsRNA injected worms. The y-axis represents the fold change in mRNA expression in the progeny of *H. bacteriophora* hermaphrodites. The mRNA levels are normalized to *nol-5* expression in the progeny of non-injected hermaphrodites (*black bar*). The green bar represents *nol-5* levels in progeny of *gfp* injected worms. The red bar represents *nol-5* levels in phenotypically wild type siblings, and the blue bar represents the *nol-5* levels in worms with empty gonads. To control for off-target effects, expression of an unrelated gene (*cct-2*) in worms with empty gonads was determined (*brown bar*). The graph was obtained by combining data from at least three independent biological replicates. Error bars indicate the standard error of the mean. Asterisks depict the statistical significance of the observed differences in unpaired, two-tailed t-tests with *P*-values < 0.001 (**)

In this study, injecting dsRNA of *Hba-cct-2* and *Hba-nol-5* in the *H. bacteriophora* hermaphrodites resulted in sterile progeny, with a noticeable lack of any germline and eggs in the adult nematodes. The observed phenotype was similar to the post-embryonic phenotype described by Ciche et al. [36], although the percentage of the sterile progeny was lower. We observed that 40 to 50 % of the progeny of *cct-2* dsRNA injected nematodes (Fig. 1b, Table 2), and 20 to 60 % of the progeny of *nol-5* dsRNA injected nematodes were sterile in three different biological replicates (Fig. 2b, Table 2). This difference in penetrance may be due to our examination of the phenotype of F1 progeny of injected nematodes, and rather than in adults derived from eggs soaked in dsRNA. For both *Hba-cct-2* and *Hba-nol-5,* dsRNA was generated using primers previously described [36]. Microinjection as the means of dsRNA delivery has never been reported in *H. bacteriophora*. Knockdown of gene transcripts resulting in visible phenotypes in progeny for both genes in three different trials confirmed that this technique can be used to reliably knockdown gene transcripts in *H. bacteriophora*.

**Table 2:** RNAi phenotypes observed in the progeny of *H. bacteriophora* worms injected with dsRNA

	Trial-1	Trial-2	Trial-3	Average
*GFP*	0 (0/53)	0 (0/95)	0 (0/127)	0 (0/127)
*nol-5 (ste)*	20 (35/173)	25 (29/113)	60 (92/152)	36 (156/438)
*cct-2 (ste)*	44 (74/165)	50 (91/179)	51 (102/198)	50 (267/542)
*dpy-13 (dpy)*	7 (3/42)	8 (2/25)	6 (3/43)	7 (8/110)
*dpy-7 (dpy)*	3 (1/26)	5 (3/53)	2 (2/98)	3 (6/177)

For each trial, percentage of observed phenotype is followed in parenthesis by total number of progeny that had the predicted phenotype over total number of progeny from the injection. Last column is the average of all the trials*. Ste* (Sterile) and *dpy* (dumpy)

**Fig. 3:**
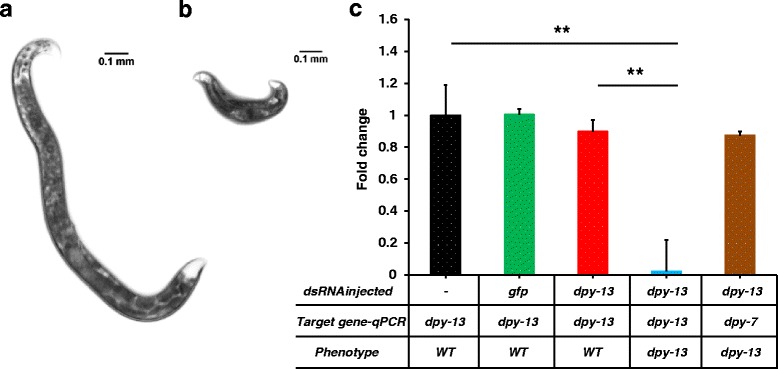
RNAi mediated phenotype and transcript changes in *H. bacteriophora* injected with *dpy-13* dsRNA. Adult *H. bacteriophora* hermaphrodites that were injected with *dpy-13* dsRNA produced progeny with dumpy phenotype. **a**. Phenotypically wild type progeny of *H. bacteriophora* worms injected with *dpy-13* dsRNA. **b**. Progeny of *H. bacteriophora* worms injected with *dpy-13* dsRNA exhibiting the dumpy phenotype. **c**. Expression of *dpy-13* gene in the progeny of *dpy-13* dsRNA injected worms. The y-axis represents the fold change in mRNA expression in the progeny of *H. bacteriophora* hermaphrodites. The mRNA levels are normalized to *dpy-13* expression in the progeny of non-injected hermaphrodites (*black bar*). The green bar represents *dpy-13* levels in progeny of *gfp* injected worms. The red bar represents *dpy-13* levels in phenotypically wild type siblings, and the blue bar represents the *dpy-13* transcript levels in phenotypically dumpy worms. To control for off-target effects, expression of an unrelated gene (*dpy-7*) in phenotypically dumpy worms was determined (*brown bar*). The graph was produced by combining data from at least three independent biological replicates. Error bars indicate the standard error of the mean. Asterisks depict the statistical significance of the observed differences in unpaired, two-tailed t-tests with *P*-values < 0.001 (**)

**Fig. 4:**
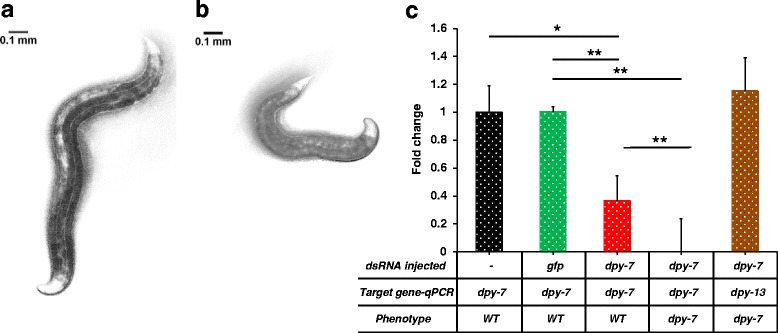
RNAi mediated phenotype and transcript changes in *H. bacteriophora* injected with *dpy-7* dsRNA. Adult *H. bacteriophora* hermaphrodites that were injected with *dpy-7* dsRNA produced progeny with dumpy phenotype. **a**. Phenotypically wild type progeny of *H. bacteriophora* worms injected with *dpy-7* dsRNA. **b**. Progeny of *H. bacteriophora* worms injected with *dpy-7* dsRNA exhibiting the dumpy phenotype. **c**. Expression of *dpy-7* gene in the progeny of *dpy-7* dsRNA injected worms. The y-axis represents the fold change in mRNA expression in the progeny of *H. bacteriophora* hermaphrodites. The mRNA levels are normalized to *dpy-7* expression in the progeny of non-injected hermaphrodites (*black bar*). The green bar represents *dpy-7* levels in progeny of *gfp* injected worms. The red bar represents *dpy-7* levels in phenotypically wild type siblings, and the blue bar represents the *dpy-7* transcript levels in phenotypically dumpy worms. To control for off-target effects, expression of an unrelated gene (*dpy-13*) in dumpy worms was determined (*brown bar*). The graph was produced by combining data from at least three independent biological replicates. Error bars indicate the standard error of the mean. Asterisks depict the statistical significance of the observed differences in unpaired, two-tailed t-tests with *P*-values < 0.01 (*) and < 0.001 (**)

We also tested two other genes, *Hba-dpy-13* and *Hba-dpy-7* that were previously described to cause a dumpy phenotype in *C. elegans*. Dumpy mutant nematodes are almost half the length of the wild type nematode. *Cel-dpy-13* and *Cel-dpy-7* encode two different collagen proteins required for proper cuticular morphology and normal body length [60, 61]. *Hba-dpy-13* was also used as a positive control for RNAi by soaking in *H. bacteriophora* [37]. Injecting *Hba-dpy-13* dsRNA resulted in dumpy-like progeny with short, chunky morphology similar to that seen in *C. elegans dpy* mutants (Fig. 3b, Table 2). Injecting *Hba-dpy-7* dsRNA also caused the progeny to be phenotypically similar to *Hba-dpy-13*RNAi (Fig. 4b, Table 2). However, the numbers of dumpy nematodes observed in both *Hba-dpy-13* and *Hba-dpy-7* dsRNA injections were significantly lower than observed with *nol-5* and *cct-2* dsRNA injections. We observed that 6 to 8 % of the progeny of *dpy-13* dsRNA injected nematodes and 2 to 5 % of the progeny of *dpy-7* dsRNA injected nematodes exhibited the dumpy phenotype across three different biological replicates (Table 2).

We observed significant variability in phenotypic expression between the genes tested. The number of nematodes with the expected phenotype ranged from 2 to 60 %. Variation in the RNAi efficacy of knockdown has been documented previously in parasitic nematodes [38, 39, 62, 63], with generally greater success in plant pathogenic nematodes than in animal pathogenic nematodes. Since the discovery of RNAi*,* the technique has been successfully used to understand the function and interaction of most genes in *C. elegans*. The inability to reliably knockdown gene expression by RNAi in parasitic nematodes is perplexing as many of the genes involved are conserved, with few exceptions [41]. Sequencing of the genome revealed that all the major classes of genes required for RNAi are present in *H. bacteriophora* [44]. Further analysis on the molecular interaction of RNAi effectors in parasitic nematodes is essential to understand the variability in RNAi efficacy.

RNAi mediated knockdown was confirmed by examining the transcript levels of the targeted genes by qRT-PCR. Knockdown of the target gene transcripts was determined by comparing transcript levels in progeny of non-injected nematodes, nematodes injected with *gfp *dsRNA, and to siblings of injected worms that were phenotypically wild type. Off-target effects of RNAi were checked by quantifying the transcript level of a non-targeted gene in nematodes with RNAi phenotype. To normalize the transcription of the genes relative to other nematodes, ribosomal protein 32 (*Hba-rpl-32*) was used as an internal control. The efficacy of all the primers used was tested before checking the transcript levels in the nematodes.

In all four RNAi knockdowns, there was a significant reduction in the mRNA levels of targeted genes when compared to both wild type and *gfp* dsRNA transcript levels. *Hba-cct-2* mRNA transcripts decreased 52 % compared to the wild type (unpaired two-tailed t-test, *t*_(4)_ = 8.82976, *P* < 0.001,), and 46 % compared to the sibling of the affected nematode (unpaired two-tailed t-test, *t*_(4)_ = 6.34638, *P* < 0.01) (Fig. 1c). We observed 58 % reduction in *Hba-nol-5* transcripts when compared to wild type nematodes (unpaired two-tailed t-test, *t*_(4)_ = 8.76645, *P* < 0.001), and 38 % reduction in the transcripts when compared to the siblings (unpaired two-tailed t-test, *t*_(4)_ = 11.25743, *P* < 0.001) (Fig. 2c). There was a reduction in the *Hba-nol-5* transcripts in the unaffected siblings compared to wild type but the reduction was not significant (unpaired two-tailed t-test, *t*_(4)_ = 0.25366, *P* = 0.812). Contrary to *cct-2* and *nol-5,* there was almost complete knockdown of the *Hba-dpy-13* and *Hb-dpy-7* transcripts in these nematodes. *Hba-dpy-13* had 97 % reduction in transcript compared to wild type animals and also sibling animals (unpaired two-tailed t-test, *t*_(4)_ = 11.37752, *P* < 0.001) (Fig. 3c), and *Hba-dpy-7* had 99 % reduction in transcript compared to wild type animals and siblings (unpaired two-tailed t-test, *t*_(4)_ = 20.28947, *P* < 0.001) (Fig. 4c). However, in the non-dumpy siblings of the dumpy nematodes there was also a 63 % reduction in *Hba-dpy-7* transcripts (unpaired two-tailed t-test, *t*_(4)_ = 4.7676, *P* < 0.01) (Fig. 4c)*.* The variation in the reduction of mRNA transcripts by dsRNA knockdown was surprising. Considering the clear RNAi phenotypes observed for *cct-2* and *nol-5* targeted animals, we predicted concomitant decreases at the transcriptional level as measured by qRT-PCR*.* This discordance suggests that there is a threshold transcript level above which the nematodes appear phenotypically wild type, and that this level may vary significantly across different genes, thereby highlighting the importance of evaluating phenotype in addition to transcript levels when determining successful gene silencing by RNAi.

## Conclusions

In this study we demonstrate that gonadal microinjection is a viable method of delivering dsRNA into *H. bacteriophora* to cause gene-specific silencing in the F1 generation. Injecting *H. bacteriophora* with dsRNA provides another strategy that researchers can deploy to generate F1 IJs with silenced genes. This provides a feasible approach to study different aspects of nematode parasitism including IJ recovery during infection, virulence factors used during infection, and the host response to parasitic infection.

## Abbreviations

dsRNA: double stranded RNA
GFP: green fluorescent protein
IJ: infective juvenile
L3: third stage larva
qRT-PCR: quantitative reverse transcriptase-polymerase chain reaction
RNAi: RNA interference
